# Chromophore-Assisted Retinal Break Detection to Manage Challenging Situations in Retinal Detachment Redo Surgery

**DOI:** 10.1155/2020/1389434

**Published:** 2020-11-30

**Authors:** Antonio Berarducci, Martina Colasante, Antonio Laborante

**Affiliations:** UOC of Ophthalmology, Head and Neck Department, IRCCS Casa Sollievo della Sofferenza Hospital, 71017 San Giovanni Rotondo, Foggia, Italy

## Abstract

**Introduction:**

The purpose of this case series is to demonstrate that subretinal blue dye injection, with and without 180-degree endolaser retinopexy, can be considered a useful tool in finding occult rhegmatogenous retinal breaks in eyes with recurrent retinal detachment. *Case Presentation*. Three patients with recurrent retinal detachment were treated between January and March 2018. In all cases, the intraoperative internal search did not demonstrate any obvious break or hole. MembraneBlue-Dual (Trypan Blue 0.15% + Brilliant Blue G 0.025% + 4% PEG) was then injected into the subretinal space using a 41-gauge cannula. The eye was rotated such that the dye was pushed through a tiny break which was causing the retinal detachment. 180-degree laser retinopexy was performed on a single eye. After silicon oil removal and absorption of the gas tamponade, retinas remained attached at three-months follow-up.

**Conclusions:**

Chromophore-assisted occult retinal break detection can be considered a useful but not risk-free surgical technique in managing some unexpected and challenging intraoperative situations.

## 1. Introduction

Finding retinal holes or breaks is a key point for a successful retinal detachment repair. In redo surgery, this goal is sometimes difficult to achieve because of the modifications of the retina and vitreous and sclera consequences of the previous surgery. Trypan Blue dye-assisted occult retinal break detection represents a relatively novel technique which can simplify identification of previously unseen retinal breaks [1] helping to achieve a better anatomical and functional success [2]. The purpose of our case study is to demonstrate that subretinal MembraneBlue-Dual injection (Trypan Blue 0.15% + Brilliant Blue G 0.025% + 4% PEG), with and without 180-degree endolaser retinopexy, can be considered an additional but not risk-free tool in managing some intraoperative challenging and complex situations.

## 2. Case Presentation

Three patients with recurrent retinal detachment were treated at the Casa Sollievo della Sofferenza Hospital between January and March 2018. All of them underwent a standard three-port pars plana vitrectomy as well as intraoperative indentation using a binocular indirect RESIGHT (Zeiss) fundus viewing system. The first patient attended vitrectomy having had previously a failed pneumoretinopexy retinal detachment repair; no holes or breaks were detected by intraoperative careful search so MembraneBlue-Dual was injected into the subretinal space using a 41-gauge cannula designed for macular translocation. Perfluorocarbon heavy liquid was then injected into the vitreous cavity displacing the subretinal fluid toward the retina periphery. The eye was rotated such that the dye was vented out of a very tiny break located just anteriorly to the preexisting corioretinal scar. A second patient with a clinical history of multiple surgeries for retinal detachment (cryobuckle+PPV+circumferential and radial retinectomy) presented with a new onset macula off retinal detachment eight months after a successful silicon oil removal. On the superior temporal quadrant was still visible an apparently well buckled and partially treated tear with no other obvious hole or break. Again, MembraneBlue-Dual was injected into the subretinal space and perfluorocarbon heavy liquid injected into the vitreous cavity displacing the subretinal fluid. The dye started leaking out of the superior temporal already buckled tear which was initially considered safe (Video 1). In the third case, a standard pars plana vitrectomy was performed on a patient who had episcleral buckling procedure four years before. The retina was stiff and the 360-degree encircling band quite anterior and tight making difficult the visualization of the retina just posterior to it. Indentation was also difficult by the presence of an extremely scarred tenon. A pocket was created in the subtenon space in order to reach the equator with very little improvement in the indentation procedure. The encircling band was then removed with very minimal gain in the peripheral view because the thinned sclera maintained the preexisting shape. MembraneBlue-Dual was again injected into the subretinal space and a BSS-perfluorocarbon heavy liquid exchange performed in order to displace the dye. Multiple areas of leakage were visible in the inferior quadrants just at the posterior edge of the indentation. As some of the retinal breaks were still not easily accessible due to the eyeball shape, a fluid-air exchange was carried out in order to improve the peripheral view. Finally, 180-degree endolaser retinopexy was performed (Video 2). After gas absorption in the first and second cases and silicon oil removal in the third one, retinas remained attached with no sign of failure at three-month follow-up.

## 3. Discussion and Conclusions

Missing retinal breaks during retinal detachment surgery is a well-recognized cause of failure in primary repairs [1]. In some cases, Lincoff's rules may address toward the hidden tear [3], but even so, occasionally, no breaks are found and this represents a very challenging surgical situation. In episcleral buckling procedures, failure occurs nearly as frequently because the buckle is inadequate, it is poorly placed, or it is too narrow or too shallow [3]. Also, sutures tend to release after some time, and the retina can detach again if tears are not properly treated at the time of the first surgery. On top of it, the sclera can keep the shape given by the preexisting encircling band even after its removal, creating additional viewing problems and subsequent risk of missing one or more breaks. In these conditions, chromophore-assisted break detection may be considered a useful surgical technique in preventing surgical failure [4, 5]. The dyes currently used for different steps in chromovitrectomy are triamcinolone acetonide, indocyanine green, infracyanine green, Brilliant Blue for internal limiting membrane identification, and Trypan Blue for the epiretinal membrane. Even if Trypan Blue has been already used for searching for retinal breaks in eye surgery [1], this technique is not free from possible downsides such as iatrogenic small-sized retinal holes or potential dye-related retinal toxicity. In our cases, a fluid-air exchange with perfluorocarbon heavy liquid protecting the posterior pole was performed in order to keep the macular area safe as much as possible; anyway, we cannot exclude that a certain amount of dye may have remained into the subretinal space. A recent study on rats' model to provide a safety profile that limits retinal toxicity while maintaining dye visibility demonstrated that Trypan Blue injection induces a dose-dependent neurotoxic effect on retinal cells and sets at 0.04% the safe concentration to be effective, avoiding retinal apoptosis [6]. Even if Brilliant Blue alone seems to be ideal because of the lower toxic profile [7] and for being lighter than water, we used MembraneBlue-Dual instead in order to enhance the visualization of any possible residual vitreous or membrane too [8, 9]. In our experience, a wide endolaser retinopexy does not represent a first line choice but can be occasionally considered to reduce the incidence of failure [10] after silicone oil removal in all situations where rhegmatogenous tears cannot be directly seen and properly marked. Similarly, even if literature suggests that retinal detachments managed with gas achieve a better visual outcome with fewer postoperative complications [11], the use of silicone tamponade associated with or without 180° inferior retinectomy appears reasonable for most patients with very complex RD associated with PVR [12] and may lead to a better anatomical success in patients who have previously undergone scleral buckle procedure for inferior retinal detachment repair compared with eyes that underwent a primary PPV [13]. Our case study represents the first one in which an association of Trypan and Brilliant Blue has been used; even if some studies suggest for the Trypan Blue alone due to no evidence of retinotoxicity [14, 15], we believe that controversy still remains around various issues, mainly potential toxicity and safety. At the same time, no other complications related with the 41-gauge subretinal injection have been reported. Anyway, even if MembraneBlue-Dual is licensed in many countries, at this stage, there is still room for further investigation to establish its safety for this specific purpose, and the current state of the art suggests that staining-assisted procedures should be performed using concentrations and volumes as low as possible [16]. For all these reasons, chromophore-assisted retinal break detection represents a method that should nowadays not be used routinely as first choice but only reserved as an extreme attempt in challenging situations with high risk of leaving breaks or holes untreated with the aim of achieving a good anatomical result and preventing further surgery.

## Abbreviations

Redo surgery: Reoperative surgery
PEG: Polyethylene glycol
PPV: Pars plana vitrectomy
BSS: Balanced salt solution
PVR: Proliferative vitreoretinopathy
RD: Retinal detachment.

## Supplementary Materials

Supplementary 1 Video 1: patient with a clinical history of multiple surgeries for retinal detachment (cryobuckle+PPV+circumferential and radial retinectomy) presented with a new onset macula off retinal detachment eight months after a successful silicon oil removal. On the superior temporal quadrant was still visible an apparently well buckled and partially treated tear with no other obvious hole or break. MembraneBlue-Dual was injected into the subretinal space through a 41 G needle and perfluorocarbon heavy liquid injected into the vitreous cavity displacing the subretinal fluid. The dye started leaking out of the superior temporal already buckled tear which was initially considered safe. The inefficient plombage was removed and laser and gas applied.

Supplementary 2 Video 2: a standard pars plana vitrectomy was performed on a patient who had episcleral buckling procedure four years before. The retina was stiff and the 360-degree encircling band quite anterior and tight making it difficult the visualization of the retina. Indentation was also difficult by the presence of an extremely scarred tenon. The encircling band was then removed with very minimal gain in the peripheral view because the thinned sclera maintained the preexisting shape. MembraneBlue-Dual was injected into the subretinal space and a BSS-perfluorocarbon heavy liquid exchange performed in order to displace the dye. Multiple areas of leakage were visible in the inferior quadrants just at the posterior edge of the indentation. A fluid-air exchange was performed in order to improve the peripheral view and a 180-degree endolaser retinopexy followed by air/oil exchange was carried out.
